# Design of a Matching Network for a High-Sensitivity Broadband Magnetic Resonance Sounding Coil Sensor

**DOI:** 10.3390/s17112463

**Published:** 2017-10-27

**Authors:** Yang Zhang, Fei Teng, Suhang Li, Ling Wan, Tingting Lin

**Affiliations:** College of Instrumentation & Electrical Engineering, Lab of Geo-Exploration Instrumentation of Ministry of Education, Jilin University, Changchun 130026, China; zhang_yang15@mails.jlu.edu.cn (Y.Z.); teng_fei@jlu.edu.cn (F.T.); suhang17@mails.jlu.edu.cn (S.L.); wanling@jlu.edu.cn (L.W.)

**Keywords:** magnetic resonance sounding, coil sensor, LC filter

## Abstract

The magnetic resonance sounding (MRS) technique is a non-invasive geophysical method that can provide unique insights into the hydrological properties of groundwater. The Cu coil sensor is the preferred choice for detecting the weak MRS signal because of its high sensitivity, low fabrication complexity and low cost. The tuned configuration was traditionally used for the MRS coil sensor design because of its high sensitivity and narrowband filtering. However, its narrow bandwidth may distort the MRS signals. To address this issue, a non-tuned design exhibiting a broad bandwidth has emerged recently, however, the sensitivity decreases as the bandwidth increases. Moreover, the effect of the MRS applications is often seriously influenced by power harmonic noises in the developed areas, especially low-frequency harmonics, resulting in saturation of the coil sensor, regardless of the tuned or non-tuned configuration. To solve the two aforementioned problems, we propose a matching network consisting of an LC broadband filter in parallel with a matching capacitor and provide a design for a coil sensor with a matching network (CSMN). The theoretical parameter calculations and the equivalent schematic of the CSMN with noise sources are investigated, and the sensitivity of the CSMN is evaluated by the Allan variance and the signal-to-noise ratio (SNR). Correspondingly, we constructed the CSMN with a 3 dB bandwidth, passband gain, normalized equivalent input noise and sensitivity (detection limit) of 1030 Hz, 4.6 dB, 1.78 nV/(Hz)^1/2^ @ 2 kHz and 3 nV, respectively. Experimental tests in the laboratory show that the CSMN can not only improve the sensitivity, but also inhibit the signal distortion by suppressing power harmonic noises in the strong electromagnetic interference environment. Finally, a field experiment is performed with the CSMN to show a valid measurement of the signals of an MRS instrument system.

## 1. Introduction

Compared to other geophysical methods, the use of MRS technology can directly determine the water content of the subsurface [1,2,3]. The method has become a competitive approach for non-invasive investigations of groundwater resources in recent years [4]. The basic principle of this method is that the transmitting loop is energized for a certain time τ by a pulse of alternating current I_0_ to stimulate a precession of the protons around the geomagnetic field. When the pulse is terminated, the protons produce a free induction decay (FID) signal with a fixed bandwidth, which depends on the pulse moment *q* = I_0_ × τ and can be detected by the pickup loop. The FID signal envelope is characterized by E_0_(*q*) and T_2_*(*q*), which are the initial amplitude and the average relaxation time of the signal, respectively [5]. After fitting the E_0_(*q*) and T_2_*(*q*) values using the processed FID signal, the water content and pore size of the rock could be determined via the inversion process [6]. MRS is a large-scale method, with the investigated volume approximated by a cube of 1.5 × d, where 100 < d < 150 m is the side of a square loop [7]. To date, the MRS technology has been successfully applied in searching for underground water resources in arid regions and detecting seawater intrusion [8,9,10,11,12]. Therefore, it is of paramount importance to obtain reliable FID signals with suitable receiving sensors for MRS applications.

Coil sensors are widely used in biomedical and geological survey instruments because of their excellent sensitivity, low fabrication complexity, robustness and low cost [13,14,15,16]. To optimize the coil sensor designs and improve the sensitivity, numerical methods have been developed [17,18,19]. An air-core induction magnetometer for detecting low-frequency fields was developed by optimizing the minimum coil size based on a specific type of amplifier and a fixed cut-off frequency [17]. Lin et al. designed a high-sensitivity cooled coil system (77 K) by optimizing the coil diameter and the conductor thickness for ultra-low-field nuclear magnetic resonance in laboratory and field conditions [18,19]. Nevertheless, the high sensitivity coil must be combined with a matching circuit that can achieve optimum performances for applications, especially in MRS, whose detection signal is on the order of nanovolts (10^−9^ V). The tuned design was initially used for the MRS coil sensor design because it can provide additional sensitivity with narrower bandwidth; however, the FID signals may be distorted through the tuned coil sensor because of the narrowband characteristics. The signal distortion is not acceptable, and this design is therefore seldom used in MRS. In the case of the non-tuned design, the sensor can obtain valid signals because of the broad bandwidth; however, the sensitivity is lower than that of the tuned design. Moreover, the FID signals sampled by the broadband sensor can be better suited for the post digital signal processing methods [20,21,22]. Lin et al. described a non-tuned coil system for the MRS application that involved minimizing the preamplifier equivalent input noise to improve the sensitivity [23]. However, the sensitivity of their MRS coil system is still lower than that of the tuned design. None of the tuned and non-tuned configurations has the advantages of both high sensitivity and broad bandwidth. Hence, our first goal is to propose a method to design a broadband MRS coil sensor with high sensitivity.

In addition, the fact that low-frequency harmonics always cause preamplifier saturation in developed areas, regardless of which configuration for the MRS coil sensor design is selected is a serious problem. Although the uses of a notch filter and “figure-eight” shaped coil are effective methods of suppressing environmental noise, including power harmonic noises [24,25], low-frequency harmonics with large amplitude prevent additional applications of the above-described MRS technique. Lin et al. proposed a real-time anti-saturation technology that provides a new method for obtaining effective FID signals [26]. This method can inhibit the post-amplifier saturation distortion, but it cannot solve the problem of preamplifier saturation. Therefore, a specific coil sensor with a novel technology for MRS applications in strong electromagnetic noise environments should be developed.

To improve the sensitivity of the system and suppress the signal distortion, we designed a matching network consisting of an LC broadband filter in parallel with a matching capacitor. Based on the characteristics of the pickup coil, the matching capacitor is selected to increase the gain in the passband of the filter and improve the sensitivity of the sensor system. The rest of this paper is organized as follows: Section 2 analyses the characteristics of the tuned and non-tuned coil sensors and the origins of the coil sensor nonlinear distortion and then introduces the design method of the matching network of the CSMN. Section 3 presents the equivalent schematic of the CSMN with noise sources. Section 4 evaluates the sensitivity of the CSMN system using the Allan variance. In Section 5, the effectiveness of the method is verified by laboratory and field experiments. Finally, Section 6 provides the conclusion of this study and recommendations for future research.

## 2. High-Performance CSMN

In this section, the advantages and disadvantages of the tuned and non-tuned configurations for the coil sensor are described in detail. The origins of the nonlinear distortion of the traditional MRS coil sensor are analysed. Finally, we introduce the design method of the matching network used for the CSMN.

### 2.1. Characteristics of the Tuned and Non-Tuned Coil Sensors

**Figure 1:**
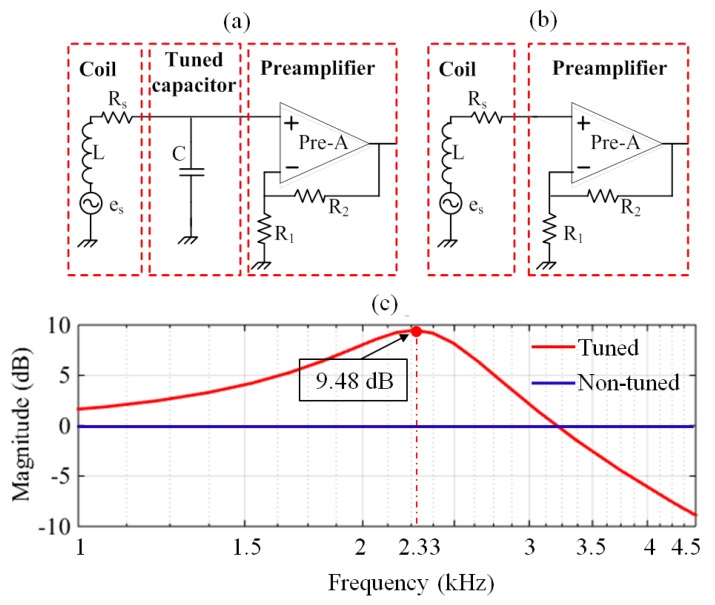
Schematics of the coil sensors with tuned and non-tuned configurations. (**a**) The tuned design; (**b**) The non-tuned design; (**c**) The amplitude-frequency characteristic curves of tuned and non-tuned configurations.

The tuned and non-tuned configurations illustrated in Figure 1 are the most widespread configurations used for the MRS coil sensor design. The schematic of the tuned design (Figure 1a) consists of three parts (i.e., coil, tuned capacitor and preamplifier). We take the resonance frequency 2330 Hz as an example to perform simulation analysis; the relevant parameters are as follows: L = 0.8 mH, Rs = 4 Ω, C = 5.8 μF and the gain of the preamplifier is 0 dB. The amplitude-frequency characteristics of the tuned design are shown in the red curve (Figure 1c). The magnitude is 9.48 dB at a resonance frequency of 2330 Hz. Therefore, this configuration has the advantages of high sensitivity and the ability to maintain the signal-to-noise ratio (SNR) with the resonance of the tuned capacitor and inductor. However, this configuration may lead to a distorted broadband FID signal. The maximum bandwidth of the signal is 105 Hz when T_2_* is 30 ms [7]. To ensure that all FID signals measured by the sensor are undistorted, the bandwidth of the sensor must be greater than 105 Hz. Inaccurate values of the tuned capacitor and inductor cause the centre frequency offset error, thus, we should leave adequate margins. Therefore, the bandwidth of the sensor is usually set to 200 Hz or more. The tuned design is rarely used for the MRS sensor because its narrow-band is lower than 200 Hz. The non-tuned design (Figure 1b) has only two parts without the tuned capacitor C; its amplitude-frequency characteristics are shown in Figure 1c (the blue curve), exhibiting a very flat full frequency bandwidth and 0 dB magnitude. This configuration, which can measure valid FID signals and facilitate the post digital signal processing because of the broad bandwidth, is currently the most commonly used structure; however, its sensitivity is lower than that in the tuned case. The sensitivity of a coil sensor directly determines the performance of an MRS instrument. In MRS applications, increasing the sensor sensitivity enables the system to detect thinner or deeper located aquifers. In addition, the preamplifier of the coil sensor using the tuned or non-tuned configurations may be saturated in strong electromagnetic noise environments. The reasons for the preamplifier saturation distortion will be analysed in the following section.

### 2.2. The Coil Sensor Nonlinear Distortion

**Figure 2:**
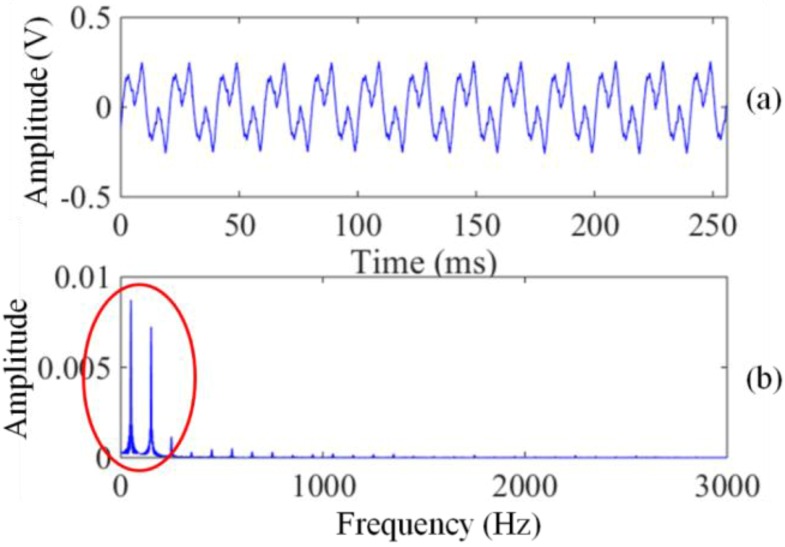
The power harmonic noise waveforms. (**a**) Time domain waveform; (**b**) Frequency domain waveform.

The power harmonic noises generated by the power line or AC power grid are the most serious interference sources in developed areas. Here, we show a group of power harmonic noises (Figure 2) collected on a lawn of Changchun City by a 100 m × 100 m square coil that is directly connected to the analogue-to-digital (A/D) acquisition card. As shown in Figure 2a, the amplitude of the harmonic noises is so high that it reaches 180 mV, especially the low-frequency harmonics (the part surrounded by the red circle in Figure 2b), whose amplitudes are hundreds of times greater than those of the other high-frequency harmonics. The low-frequency harmonics with large amplitude is one of the main reasons for the coil sensor nonlinear distortion.

**Figure 3:**
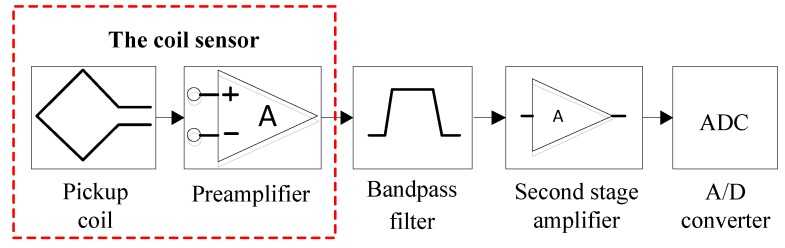
The traditional MRS data acquisition system.

Moreover, we find that the circuit structure of the traditional MRS data acquisition system with the coil sensor (the part surrounded by the red dotted line in Figure 3) is another reason for the coil sensor nonlinear distortion. As shown in Figure 3, the bandpass filter after the preamplifier is ineffective because the distortion of the coil sensor has already occurred.

### 2.3. Design of the Matching Network

**Figure 4:**
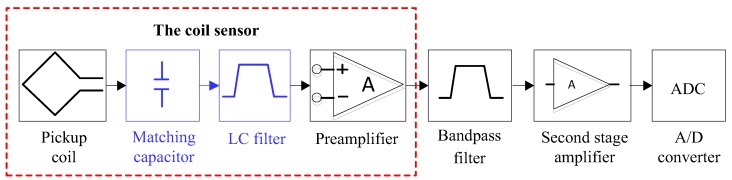
The new MRS data acquisition system.

To improve the sensitivity of the broadband coil sensor and suppress the low-frequency harmonics, we propose a new circuit structure of the MRS data acquisition system, as shown in Figure 4. The CSMN (the part is surrounded by the red dotted line in Figure 4) is designed by adding an LC filter with a matching capacitor between the pickup coil and the preamplifier compared with the traditional broadband non-tuned coil sensor in an MRS instrument (Figure 3). The matching capacitor connected in parallel with an LC filter (the blue part in Figure 4) composes the matching network as the core of the new sensor.

We consider an LC filter with a bandwidth of 1500 Hz and a centre frequency of 2330 Hz as an example to introduce the matching network’s design method, which includes the three following steps:

(1) The topology of the LC bandpass filter

**Figure 5:**
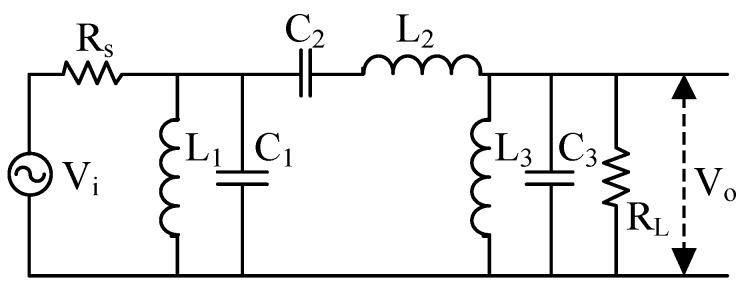
π type 3 order LC passive bandpass filter.

To effectively suppress the low-frequency harmonics, the gain of the LC filter at 500 Hz is less than −30 dB, and the gain at 100 Hz is less than −50 dB. In addition, to simplify design and reduce cost, we use the topology of π-type 3-order LC passive bandpass filter, as shown in Figure 5. (Note that other circuit structures can also be selected, such as T-type 5-order filter, as long as the design indicators can be met.)

(2) The approximate function and the calculation of the parameters of the filter

To avoid FID distortion, we choose the Butterworth function, which has the advantage of relative flatness in the passband. As mentioned above, the selected bandwidth BW = 1500 Hz and centre frequency F_0_ = 2330 Hz of the LC filter leads to the −3 dB low cutoff frequency and high cutoff frequency of F_L_ = F_0_ − BW/2 = 1580 Hz and F_H_ = F_0_ + BW/2 = 3080 Hz, respectively. The relative centre frequency and the quality factor can be calculated as F_m_ = (F_L_ × F_H_) ^1/2^ = 2206 Hz and Q = F_m_ /BW = 1.47, respectively. From Table 1, we can find the normalized values K_1_ = K_3_ = K = 1, and K_2_ = 2. In addition, to reduce the thermal noise of the preamplifier, we choose R_L_ = R = 50 Ω. According to the normalized values and the empirical formula, we calculate the value of each parameter as follows: L_1_ = L_3_ = R/(2πKQF_m_) = 2.46 mH, L_2_ = QRK_2_/(2πF_m_) = 10.6 mH, C_1_ = C_3_ = QK/(2πRF_m_) = 2.12 μF and C_2_ = 1/(2πRQK_2_F_m_) = 0.491 μF.

**Figure 6:**
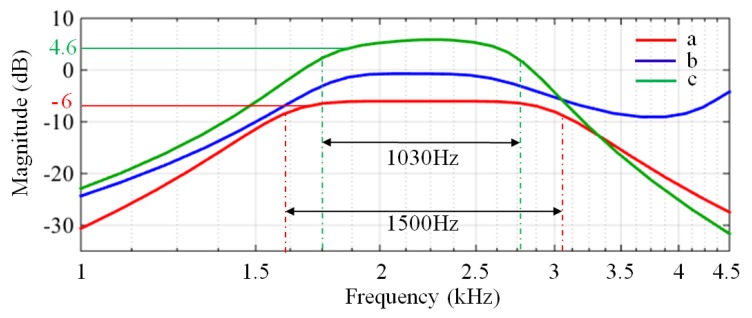
The amplitude-frequency characteristic curves of the filter. (**a**) The LC filter; (**b**) The LC filter connected with the pickup coil; (**c**) The LC filter connecting in parallel with a matching capacitor and the pickup coil.

The amplitude-frequency characteristic curve of the LC filter designed is shown in Figure 6 (the red curve). The test results show that the LC filter can meet the design indicators with a gain at 500 Hz of −53.5 dB, a gain at 100 Hz of −95.4 dB, a bandwidth of 1580 Hz and a very flat passband. However, the gain within passband of the filter of −6.02 dB is problematic because the FID signal will be attenuated by half through the filter, thereby increasing the difficulty of signal detection (even reducing the detection depth). The solution for this attenuation problem will be introduced in the next step.

(3) Selection of the matching capacitor

As an example, we take a 100 m × 100 m square pickup coil with one turn, which can be equivalent to a series circuit of an inductor (L = 0.8 mH) and a resistor (Rs = 4 Ω). The amplitude-frequency characteristic curve of the LC filter connected with the pickup coil is shown in Figure 6b (the blue curve). It can be seen that the curve is obviously distorted and the gain in the passband of the filter is attenuated.

**Figure 7:**
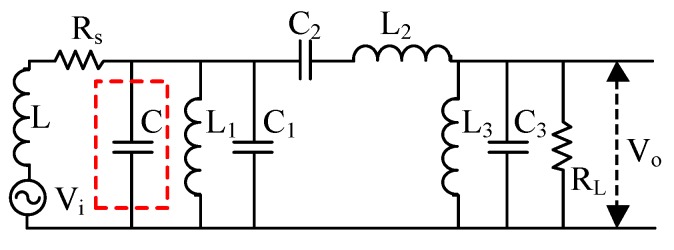
The circuit structure of the LC bandpass filter connecting a matching capacitor.

To solve the above problem, our idea is to connect a matching capacitor in parallel with the LC filter to cancel the influence of the pickup coil on the post-stage circuit. The new passive filter connected to the matching capacitor and the pickup coil is shown in Figure 7 (the matching capacitor is located within the red dotted line). We find that the effect of the pickup coil on the amplitude-frequency characteristic can be cancelled when the reactance of the equivalent inductor and the matching capacitor is zero at the centre frequency (F_0_ = 2330 Hz). The value of the matching capacitor C can be calculated as:

(1) $$C=\frac{1}{{\left(2\pi F_{0}\right)}^{2}\times L}=5.8\mu F$$

The amplitude-frequency characteristic curve of the new passive filter connected to the matching capacitor and the pickup coil is shown in Figure 6 (the green curve). The gain of the new filter at 500 Hz and at 100 Hz is −40 dB and −73.8 dB, respectively, and the gain in the passband is 4.6 dB, which eliminates the attenuation. The bandwidth of the filter has changed, and the −3 dB low and high cutoff frequencies are 1730 Hz and 2760 Hz, respectively. In fact, the bandwidth (BW = 1030 Hz) is adequate for detecting the FID signals in most areas. Moreover, according to actual needs, we can design a larger or smaller bandwidth based on the above design method.

**Figure 8:**
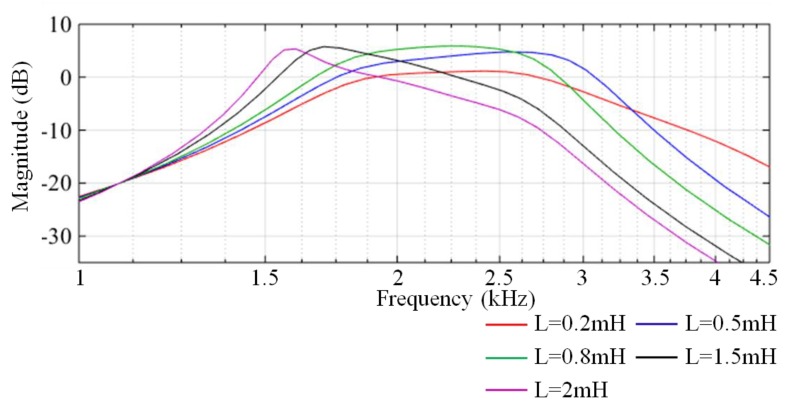
Monte Carlo analysis of the equivalent inductance of the pickup coil.

**Figure 9:**
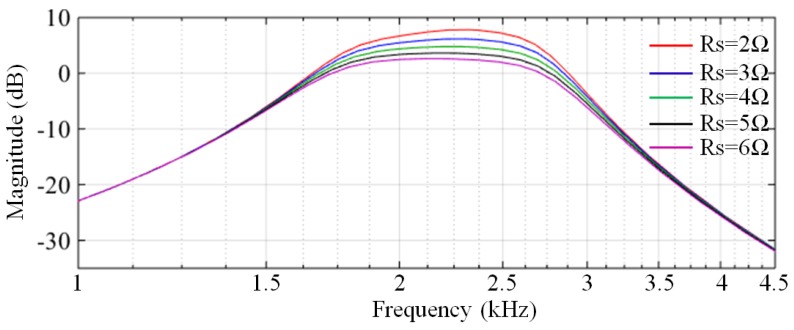
Monte Carlo analysis of the equivalent resistance of the pickup coil.

Note that the matching network is sensitive to the parameters of the pickup coil. The equivalent inductance and equivalent resistance of the pickup coil have a great influence on the performance of the matching network. We analyse the characteristics of the matching network with different inductances and different resistances via Monte Carlo simulation. The Monte Carlo analysis results of the equivalent inductance (Figure 8) show that only the amplitude-frequency characteristics of the filter with L = 0.8 mH (the green curve) is correct, whereas the results of other values are distorted. The Monte Carlo analysis results of the equivalent resistance (Figure 9) show that the gain in the passband increases as the resistance reduces.

Therefore, we designed a new coil sensor with a matching network. To make effective use of the CSMN, the value of the matching capacitor by using formula 1 for different equivalent parameter of the pickup coil should be adjusted.

## 3. Equivalent Circuit of the CSMN with Noise Sources

**Figure 10:**
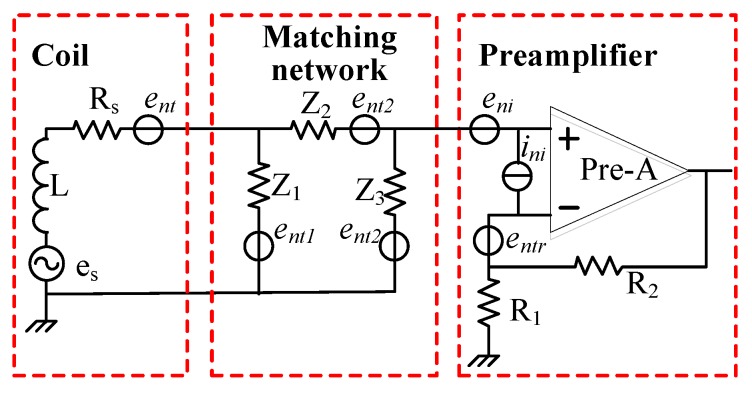
The proposed CSMN circuits with equivalent noise sources.

In this section, equivalent circuits of the CSMN with the noise source are analysed. Figure 10 presents the schematic diagrams of the CSMN with noise sources.

### 3.1. The Proposed CSMN

As shown in Figure 10, the CSMN consists of three stages, namely, the air core coil, a matching network and a low noise preamplifier. The air core coil is used to effectively detect the FID signal, which is indicated by the variations of the magnetic field. A low noise preamplifier is designed to achieve a high gain and a high noise rejection ratio. Easy gain control is realized by changing the value of two resistors R_1_ and R_2_, which can amplify the low amplitude signal to a few nV. The matching network consisting of an LC filter and a matching capacitor is shown in Figure 4. As mentioned above, the matching network circuit design makes it possible to detect all FID signals within the passband filter bandwidth without distortion.

Considering the gain *G*(*f*) of the matching network, the noise floor of the setup is used to estimate the CSMN SNR, such that:

(2) $$SNR(f)=e_{s}(f)G(f)/e_{n}(f)$$

where *e_s_*(*f*) is the output signal induced in the coil, and *e_n_*(*f*) denotes the total equivalent voltage noise at the input of the preamplifier. This total noise is calculated from all the individual noise sources depicted in Figure 10 and consists of three components (i.e., input voltage noise *e_ni_*, input current noise *i_ni_*, and Nyquist noise *e_t_* = (4*kTR_tot_*)^1/2^ of all resistors in Figure 10) [17,27]. We assume these three noise components to be uncorrelated. All these noise contributions can be combined to obtain *e_n_* as expressed below.

(3) $$e_{n}=\sqrt{e_{ni}^{2}+(i_{ni}Z_{i}{)}^{2}+(4kTR_{tot}{)}^{2}}$$

where *Z_i_* is the impedance of all resistors that input current noise flows through, *T* is the environmental temperature of resistance *R_tot_*, and *k* is the Boltzmann factor $\left(1.38\cdot 10^{-23}\;W\cdot s/K\right)$. *e_ni_* and *i_ni_* represent the input voltage noise and the input current noise of the preamplifier, respectively.

In summary, the values for the individual components of the CSMN with the matching network can be calculated as:

(4) $$i_{ni}Z_{i}=\sqrt{{\left(i_{ni}Z\right)}^{2}+(i_{ni}R_{eq}{)}^{2}},$$

(5) $$Z=(((j\omega L+R_{s})\|Z_{1})+Z_{2})\|Z_{3},$$

(6) $$Z_{1}=j\omega L_{1}\|\frac{1}{j\omega (C+C_{1})},$$

(7) $$Z_{2}=j\omega L_{2}+\frac{1}{j\omega C_{2}},$$

(8) $$Z_{3}=R_{L}\|j\omega L_{3}\|\frac{1}{j\omega C_{3}},$$

(9) $$R_{eq}=R_{1}\|R_{2},$$

where ω is the angular frequency, ω = 2π*f*.

The Nyquist noise *e_t_* produced by all resistors included can be obtained as:

(10) $$T_{n}=\sqrt{{\left(\frac{G\sqrt{4kTR_{s}}}{R_{s}+j\omega L}Z\right)}^{2}+{\left(\frac{\sqrt{4kTR_{L}}}{R_{L}}Z\right)}^{2}+{\left(\sqrt{4kTR_{eq}}\right)}^{2}}$$

The above electrical parameters of the CSMN are analysed, respectively. The CSMN with low input noise can be achieved by adopting an operational amplifier with reasonable input voltage noise and input current noise.

### 3.2. Optimization for Electrical Specification of the Preamplifier

**Figure 11:**
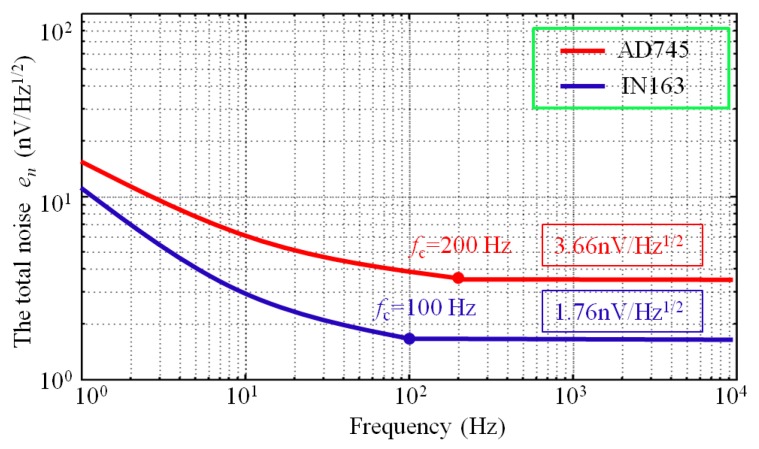
The total noises of two sensors calculated using AD745 and INA163.

To optimize the CSMN, we investigated the noise *e_n_* contributions of the CSMN by using different operational amplifiers. Two low noise operational amplifiers (i.e., INA163 and AD745) are adopted. INA163, which is a low-noise, low-distortion instrumentation amplifier, has a very low input voltage noise *V_n_* = 1 nV/(Hz)^1/2^ @ 2 kHz and a relatively high input current noise *I_n_* = 0.8 pA/(Hz)^1/2^ @ 2 kHz. Conversely, AD745, which is an ultralow-noise, high-speed preamplifier has a slightly higher input voltage noise *V_n_* = 2.9 nV/(Hz)^1/2^ @ 2 kHz and a relatively lower input current noise *I_n_* = 6.9 fA/(Hz)^1/2^ @ 2 kHz than INA163. The two resistors *R*_1_ and *R*_2_ are set to 1 Ω and 100 Ω, respectively. All other electrical parameters are set to the values calculated in Section 2, as shown in Table 2. The total noise *e_n_* of the CSMN by using INA163 and AD745, respectively, are calculated, as shown in Figure 11.

The whole frequency bandwidth can be divided into two regions (i.e., 1/*f* and broadband regions) [28,29]. In the 1/*f* region, the total noise exhibits the characteristics of a 1/*f* slope that decreases as frequency increases and reaches the minimum value at the corner frequency. For AD745, the corner frequency is 200 Hz, which is higher than that of IN163 (i.e., 100 Hz). The broadband region corresponds to higher frequencies than the corner frequency, in which the total noise is flat and is the mainly usable bandwidth of the MRS instrument system. In the broadband region, the total noise of AD745 is as low as 3.66 nV/(Hz)^1/2^, and IN163 produces a smaller noise (1.76 nV/(Hz)^1/2^) than that of AD745. Accordingly, IN163 is chosen as the preamplifier of the CSMN.

**Figure 12:**
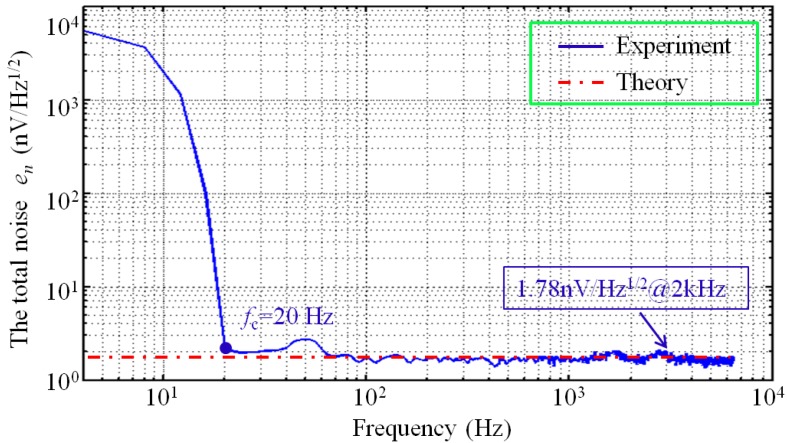
The total noise *e_n_* of the proposed CSMN.

To measure the total noise *e_n_* of the CSMN, an inductor is designed and connected to a resistor to simulate a pickup coil, whose equivalent inductance and resistance are 0.8 mH and 4 Ω, respectively. The CSMN is developed by adopting the matching network mentioned above and the preamplifier IN163. The dynamic signal analyser 35670A is employed in the shielding room to minimize interference outside the room. Figure 12 displays the results. The total noise *e_n_* in the 1/*f* region is very high. The 1/*f* noise sharply decreases with the increase in frequency until it reaches 20 Hz and then decreases gradually in the regions between 20 Hz and 100 Hz. The total noise *e_n_* tends to be stable after the corner frequency 100 Hz and is as low as 1.78 nV/(Hz)^1/2^ in the broadband region. The agreement between the experimental and theoretical calculated results shows the reliability of the CSMN model with noise sources.

We evaluate the bandwidth noise of the CSMN at one stacking time according to the total noise floor measured above. The value equivalent to the square root of the integration of the noise floor in 3 dB bandwidth is described as follows:

(11) $$e_{n}{}_{\_band}=\sqrt{\int_{B}e_{n}{}^{2}}$$

where B is the bandwidth. The minimum bandwidth of the system is 200 Hz, which allows one to obtain the valid FID signals [7,30]. Consequently, the value equivalent of the bandwidth noise is calculated as 25.2 nV, which represents the amplitude of this sensor system noise when the bandwidth is 200 Hz. We assume that this noise is white noise with a Gaussian distribution that reduces to (N_a_)^1/2^ times when averaged N_a_ times (i.e., N_a_ stacking times). In addition, we utilize the Allan variance to describe the optimum averaging time of the proposed sensor system. The sensitivity of the CSMN can be redefined and predicted based on the Allan variance and the SNR in the next section.

## 4. Sensitivity of the CSMN

Signal averaging is usually an effective method for improving the sensitivity. In terms of a perfectly stable system, the signal could be averaged infinitely in theory, and infinite averaging should lead to extremely sensitive measurements. However, all real systems are stable only for a limited time because of temperature drifts, etc. Consequently, every real system has an optimum averaging time that can be described using the Allan variance [31,32].

We utilize the Allan variance to analyse the stability of the new MSR instrument with the CSMN leading to a detection limit (i.e., the sensitivity) for the FID signal. For a formal description of the stability of our MRS instrument, we assume a recorded set of *N* time-series data *x*. The *N* elements of the time-series data can be divided into *M* subsets containing *k* elements, where *M* = *N*/*k*. Next, each of these subgroups is averaged, and if *i* denotes the subgroup number, then the subgroup average value *A_i_(k)* can be calculated:

(12) $$A_{i}(k)=\frac{1}{k}\sum_{m=1}^{k}x_{(i-1)k+m},i=1,\ldots ,M$$

The Allan variance is given by:

(13) $$\sigma_{A}^{2}(k)=\frac{1}{2M}\sum_{i=1}^{M}\left(A_{i}(k)-A_{i}(k\right){)}^{2}$$

**Figure 13:**
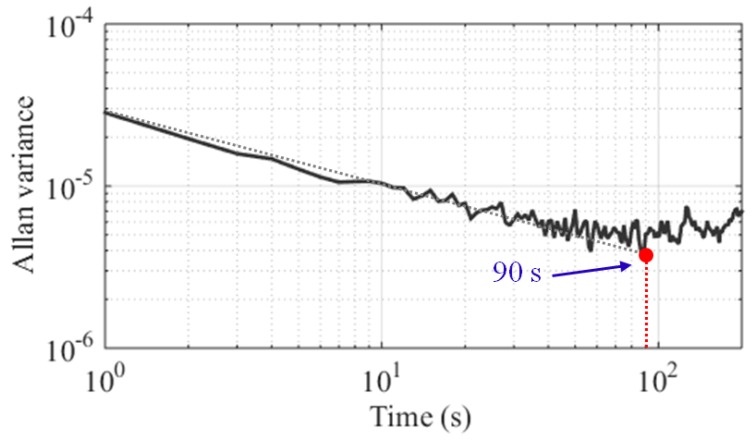
Allan variance of the new system as a function of integration time. The grey and red dashed lines are used to obtain the maximum available averaging time of 90 s.

To obtain the optimum averaging time of our new system by Allan variance, 1000 continuous tests (*N* = 1000) and 1 second acquisition intervals are used. In Figure 13, the black curve is the Allan variance plot as a function of the integration time. The minimum of the Allan variance representing the optimum integration time of 90 s is determined by the intersection of the grey dashed line and the red dashed line.

The Allan variance is proportional to the sensitivity measurement of a given system; therefore, we can use the Allan variance plot and SNR to redefine and predict the sensitivity of the MRS instrument. First, the broadband noise *e_n-band_* of the sensor is calculated or measured over the effective bandwidth. Second, we use the Allan variance to determine the optimum averaging time N_a_. Third, the new broadband noise is reduced to *e_n-band_*/(N_a_)^1/2^ after N_a_ averaging times. Finally, given that the signal is valid in MRS application when the SNR is greater than 2, the sensitivity of a sensor is calculated as follows:

(14) $$e_{s}=2e_{n\_band}/\sqrt{N_{a}\;}$$

In addition, the higher sensitivity of the CSMN system with the LC filter can be obtained according to the formula (2) with the gain G (4.6 dB) in the passband of the matching network.

(15) $$e_{s\_CSMN}=2e_{n\_band}/\sqrt{N_{a}\;}/G,$$

Therefore, the sensitivity measurement is 3.12 nV when the optimum averaging number and the broadband noise are 90 and 25.2 nV, respectively.

## 5. Experiment

In this section, the performance of the CSMN that could inhibit the coil sensor nonlinear distortion and the high sensitivity is first verified in the laboratory. Finally, a field experiment is performed to show the valid measurement of the FID signals with the CSMN.

### 5.1. Experiment for Suppressing the Signal Distortion

To verify the performance of the CSMN system, a comparative experiment between the new MRS data acquisition system (Figure 4) based on the CSMN and the traditional MRS data acquisition system (Figure 3) was conducted in laboratory under the same conditions. The technical indices of the systems are as follows. (1) The new MRS system: the LC filter bandwidth is 1030 Hz, the gain within the passband is 4.6 dB, the preamplifier (INA163) gain is 40 dB, the active bandpass filter has a bandwidth of 1000 Hz, and the overall magnification of the new system is 4000. (2) The traditional system: the overall magnification of the system is 4000, with a preamplifier (INA163) gain of 40 dB and an active bandpass filter with bandwidth of 1000 Hz.

**Figure 14:**
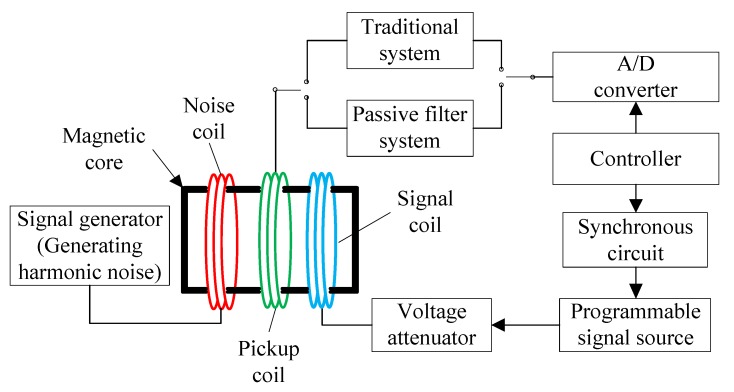
Schematic diagram of the test in the laboratory.

Both systems were tested in the laboratory according to the schematic diagram shown in Figure 14. A programmable signal source (AFG3021B, Tektronix, Johnston, OH, USA) is used to generate an exponential decay signal with E_0_ = 500 mV, T_2_* = 150 ms and *f_L_* = 2330 Hz. The programmable signal source is connected to a 2 × 10^6^ times voltage attenuator. Hence, a simulated FID signal with a 250 nV amplitude is produced in the signal coil (the blue one in Figure 14). In addition, a signal generator (HDG2102B, Qingdao Hantek Electronic Co., Qingdao, China) is connected to the noise coil (the red coil in Figure 14) to produce a low-frequency power harmonic noise with fixed amplitude at 500 Hz. The pickup coil (the green coil in Figure 14) is closely placed in parallel with the signal coil and the noise coil. As the three coils have the same configuration, an integrated signal that contains a decaying FID signal with E_0_ = 250 nV, T_2_* = 150 ms, *f_L_* = 2330 Hz and a low-frequency power harmonic noise of 500 Hz can be induced. The inductance of every coil is 0.8 mH, and the pickup coil is connected in series with a 4 Ω resistor to simulate a 100 m × 100 m receiving coil with one turn. The programmable signal source is set to external trigger mode, and the signal generator is set to continuous operation mode. As shown in Figure 14, the specific working process is as follows: the controller triggers the programmable signal source to generate the simulated signal through the synchronous circuit; meanwhile, the A/D converter is activated and acquires data.

We adjust the signal generator so that the amplitude of the 500 Hz noise is 10 mV and then perform the comparative experiment between the new system and the traditional system using the method described above. Both groups are stacked 90 times. Similarly, the amplitude of the noise is adjusted to 150 mV, 200 mV and 300 mV for the experiments.

The key parameters E_0_ and T_2_* are extracted after the experiments, as shown in Table 3. The traditional system and the new system can obtain the effective E_0_ and T_2_* when the amplitude of the noise is 10 mV or 150 mV. In addition, the signal accuracy sampled by the new system is higher than that of the traditional system because of the lower background noise and higher sensitivity of the new coil sensor system.

**Figure 15:**
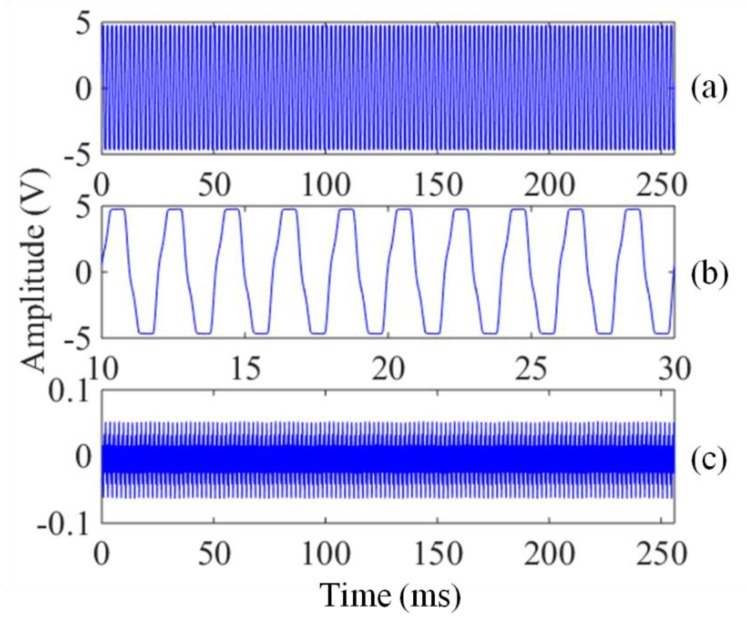
Time-domain signals after the preamplifier. (**a**) Signal sampled by using the traditional system; (**b**) Enlarged saturation signal from 10 to 30 ms in Figure 15a; (**c**) Signal sampled by using the new system with the CSMN.

However, the traditional system cannot obtain the effective E_0_ and T_2_* when the amplitudes of the noise are 200 mV and 300 mV. In contrast, the new system can still extract the effective E_0_ and T_2_* at the noise amplitude of 200 mV and 300 mV. When the noise amplitude is 200 mV, the data after the preamplifiers are sampled, as shown in Figure 15. The time-domain signal (Figure 15a) sampled with the traditional system is distorted because of preamplifier saturation. To obtain a better view of the saturation region, the signal from 10 to 30 ms in Figure 15a is enlarged in Figure 15b In contrast, the time-domain signal sampled with the new system is valid without preamplifier saturation, as shown in Figure 15c.

When the noise amplitude is greater than a certain value, the preamplifier of the traditional system will saturate. This value is determined by the maximum output voltage of the preamplifier U_0_ and the preamplifier gain A. The value can be calculated as V_max_ = U_0_/A. The maximum output voltage of the preamplifier is 4.76 V, and the preamplifier gain is 30 times. Therefore, when the noise amplitude is greater than 158.7 mV, the traditional system cannot obtain the effective data with the signal distortion, whereas the new system can obtain valid data because the CSMN can effectively suppress the noise.

### 5.2. Experiment for the Sensitivity of the CSMN

To verify the sensitivity performance of the CSMN system, another experiment on the CSMN system was conducted in the shielding room. In this experiment, we used the same technical indices and schematic diagram (Figure 14) as those of the experiment described above. The programmable signal source is adjusted to generate an exponential decay signal with E_0_ = 30 mV, T_2_* = 150 ms and *f_L_* = 2330 Hz. In addition, a simulated FID signal with a 3 nV amplitude is produced in the signal coil through the 1 × 10^7^ times voltage attenuator. In this experiment, we did not use the signal generator. Similarly, as these two coils have the same configuration, an FID signal with E_0_ = 3 nV, T_2_* = 150 ms, and *f_L_* = 2330 Hz can be induced by the pickup coil. The CSMN system was tested in the same manner as before, and the number of stacking is 90 times.

**Figure 16:**
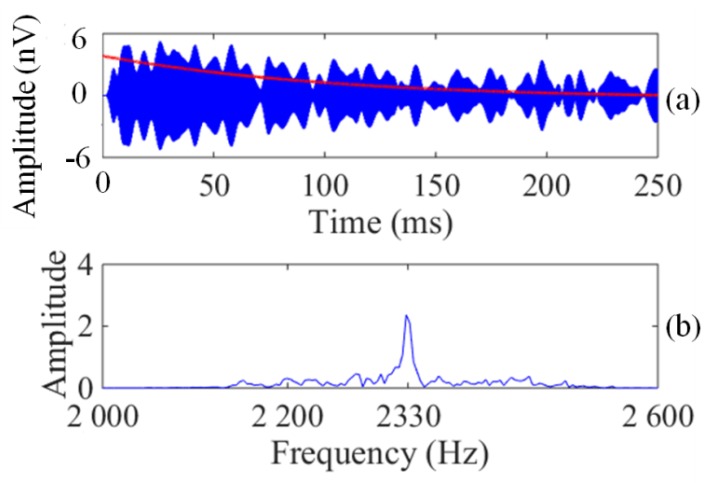
The results for the CSMN system. (**a**) Time domain signals (the blue curves) and envelope fitting curve (the red one); (**b**) Frequency domain signals.

The results obtained by the CSMN system after a digital bandpass filter and 90 stacks are shown as Figure 16. The relaxation FID signal allows the signal to be observed in both the time (Figure 16a) and frequency domains (Figure 16b) using the CSMN system. An envelope fitting curve with the exponential decay is found (the red line in Figure 16a), and the fitting E_0_ is 3.5 nV. The agreement between the experimental and theoretically calculated results shows that the CSMN system can effectively detect the signal, provided its amplitude is greater than 3 nV.

### 5.3. Field Experiment

To further verify the performance of the CSMN system, a field experiment was conducted in a suburb of Changchun city in China. A 100 m × 100 m square receiving coil with one turn is laid at a field site near a brickyard that generates a variety of large amplitude noises. In addition, a borehole exists near the field site, on which pumping tests were conducted. Ten different *q* values ranging from 0.2 to 4.5. As were employed, and 90 time stacks were used in the experiment.

**Figure 17:**
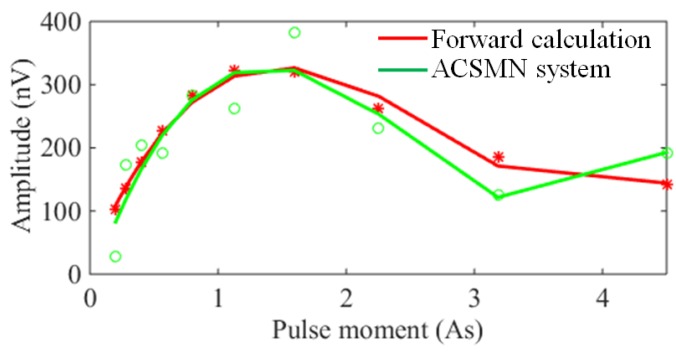
E_0_ vs. *q* curves extracted by the forward calculation and the CSMN system.

The detailed drilling data shows that three aquifers can be observed at a depth of 13–25 m, 37.5–39 m and 45–50 m. The water content of the three aquifers is 5.0%, and the water content of the other underground media layers is 1%. Based on this drilling data, 10 values of E_0_ vs. *q* marked by red stars and the corresponding fitting curve (the red curve) are given by the forward calculation in Figure 17. The values of E_0_ extracted from the data sampled by the CSMN system are marked by a green circle and the corresponding fitting curve of E_0_ vs. *q* (the green one) in Figure 17. This fitting curve of E_0_ vs. *q* is in good agreement with that of the forward calculation. The results of the field experiment show the satisfactory performance of the designed CSMN in accurately measuring the FID signals of an MRS system.

## 6. Conclusions and Prospects

For MRS applications, the proposed CSMN was designed to improve the sensitivity of the system and prevent signal distortion by adding an LC filter with a matching capacitor between the pickup coil and the preamplifier. The CSMN integrates the advantages of the tuned and non-tuned configuration sensors. The basic working principle and design method of this CSMN were elaborated, and a generalized electrical model was introduced. The schematic diagrams of the CSMN with noise sources were analysed correspondingly. To minimize the noise floor and maximize the SNR, the operational amplifier of the preamplifier was designed and optimized. To unify the capability of the MRS system detecting the minimum signal, we provided a more rigorous definition of the sensitivity of the MRS instrument using the Allan variance. Compared with the traditional system, the results of the laboratory indicated that the proposed sensor can have a greater ability at suppressing the low frequency noise with a higher sensitivity. Finally, a field experiment was performed using a fabricated sensor to demonstrate the reliability of an MRS system based on the designed matching network. Accordingly, the CSMN not only improves sensitivities of the MRS instrument but also inhibits the signal distortion by suppressing power harmonic noises in the strong electromagnetic interference environment. Moreover, the specifications of the sensor can be changed in accordance with the requirements of the local MRS exploration on the basis of the described optimization procedures. In our future work, the noise sources of the sensor (e.g., coloured noise in the developed areas) and a denoising algorithm will be explored.

## Figures and Tables

**Table 1:** Normalized values of π type 3 order LC filter.

π	Bessel	Butterworth	Chebyshev
K_1_	0.337	1.000	1.633
K_2_	0.971	2.000	1.436
K_3_	2.203	1.000	1.633

**Table 2:** Electrical parameters of the proposed CSMN.

Parameters	*L*	*Rs*	*C*	*L*_1_	*C*_1_	*L*_2_	*C*_2_	*L*_3_	*C*_3_	*R_L_*	*R*_1_	*R*_2_
Value	0.8	4	5.8	2.46	2.12	10.6	0.491	2.46	2.12	50	1	100

The unit of inductance (mH); the unit of capacitance (μF); the unit of resistance (Ω).

**Table 3:** The experimental results in laboratory.

Noise Amplitude	Traditional System		New System	
	E_0_ (nV)	T_2_* (ms)	E_0_ (nV)	T_2_* (ms)
10 mV	245.2	155.6	249.3	152.6
150 mV	243.8	154.3	248.5	153.8
200 mV	*	*	246.9	155.2
300 mV	*	*	246.3	153.4

Remark: The * means that the valid key parameters can’t been extracted because of the preamplifier saturation.
